# No Magic Bullet: Tungsten Alloy Munitions Pose Unforeseen Threat

**Published:** 2005-06

**Authors:** Charles W. Schmidt

In response to concerns about the human and environmental health effects of materials used to produce munitions, countries including the United States have begun replacing some lead- and depleted uranium–based munitions with alternatives made of a tungsten alloy. But this solution may not be the “magic bullet” it was once envisioned to be. Researchers from the Armed Forces Radiobiology Research Institute and the Walter Reed Army Institute of Research now report that weapons-grade tungsten alloy produces aggressive metastatic tumors when surgically implanted into the muscles of rats **[*EHP*** **113:729–734]**. These findings raise new questions about the possible consequences of tungsten exposure, and undermine the view that tungsten alloy is a nontoxic alternative to depleted uranium and lead.

In the study, male F344 rats were implanted with pellets in each hind leg, an exposure protocol that mimicked shrapnel wounds received in the field. The rats were split into four treatment groups: a negative control implanted with 10 pellets of tantalum (an inert metal), a positive control implanted with 10 pellets of nickel (a known carcinogen), a high-dose group implanted with 10 pellets of tungsten alloy, and a low-dose group implanted with 4 pellets of tungsten alloy and 16 pellets of tantalum. The alloy used in this research was the same as that used in weapons: 91.1% tungsten, 6.0% nickel, and 2.9% cobalt.

By 6 months after implantation all the rats in the high-dose, low-dose, and positive control groups had developed leg tumors. None of the rats in the negative control developed tumors, and all survived beyond 12 months with no apparent health effects. All remaining rats were sacrificed at 24 months.

At sacrifice, blood samples were assessed for a range of hematologic parameters. The high-dose group exhibited statistically significant increases in levels of white blood cells, red blood cells, hemoglobin, and hematocrit as compared to the low-dose and control groups.

The rats also underwent a pathology exam, and tissues were collected for histology. Whereas the tantalum pellets in the low-dose group were surrounded by normal tissue, all of the tungsten alloy and nickel pellets were surrounded by tumors. Tumors in the tungsten alloy–treated animals metastasized to the lung. Histology further indicated that tungsten alloy pellets were surrounded by invasive neoplastic cells that had infiltrated into skeletal muscle tissue. No metastasis was observed in the positive controls.

Organ measurements identified significant increases in both spleen and thymus body-to-weight ratios in the high-dose group only. Both these organs are components of the immune system, leading the authors to suggest that embedded tungsten alloy may be immunotoxic at certain concentrations.

The authors write that the amounts of cobalt (a suspected human carcinogen) and nickel in the tungsten alloy material likely were too small to produce the effects seen in the two groups implanted with the alloy. However, they do cite recent evidence indicating that the combination of these metals may produce synergistic effects. The biological mechanism by which embedded tungsten alloy produces tumors is unclear, they add, and warrants further study.

## Figures and Tables

**Figure:**
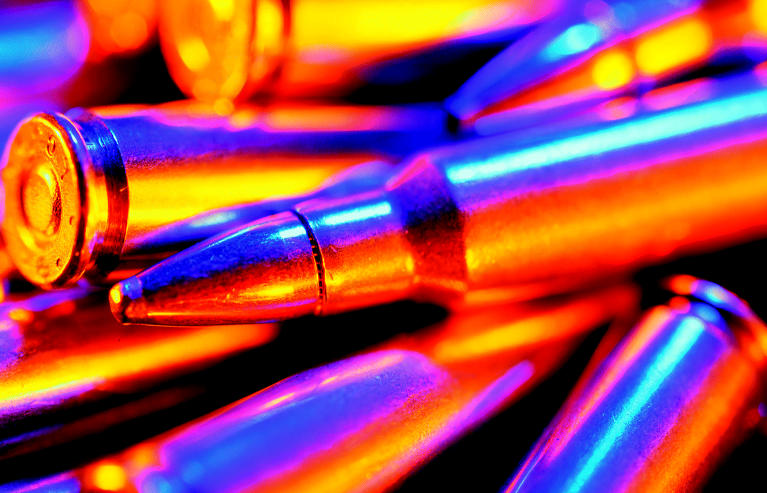
**“Better” bullets?** New data show that tungsten alloy, used in munitions in hopes it would be an environmentally friendlier alternative to lead and depleted uranium, causes tumors in animals.

